# Left ventricular abscess with native mitral valve endocarditis diagnosed by cardiac computed tomography

**DOI:** 10.1093/ehjcr/ytaf165

**Published:** 2025-04-05

**Authors:** Ryosuke Honda, Yuya Masuda, Shoichi Matsukage, Akiyoshi Ogimoto

**Affiliations:** Division of Cardiology, Uwajima City Hospital, Gotenmachi 1-1, Uwajima, Ehime 798-8510, Japan; Division of Hematology, Uwajima City Hospital, Gotenmachi 1-1, Uwajima, Ehime 798-8510, Japan; Division of Pathology, Uwajima City Hospital, Gotenmachi 1-1, Uwajima, Ehime 798-8510, Japan; Division of Cardiology, Uwajima City Hospital, Gotenmachi 1-1, Uwajima, Ehime 798-8510, Japan

## Case description

**Figure 1:**
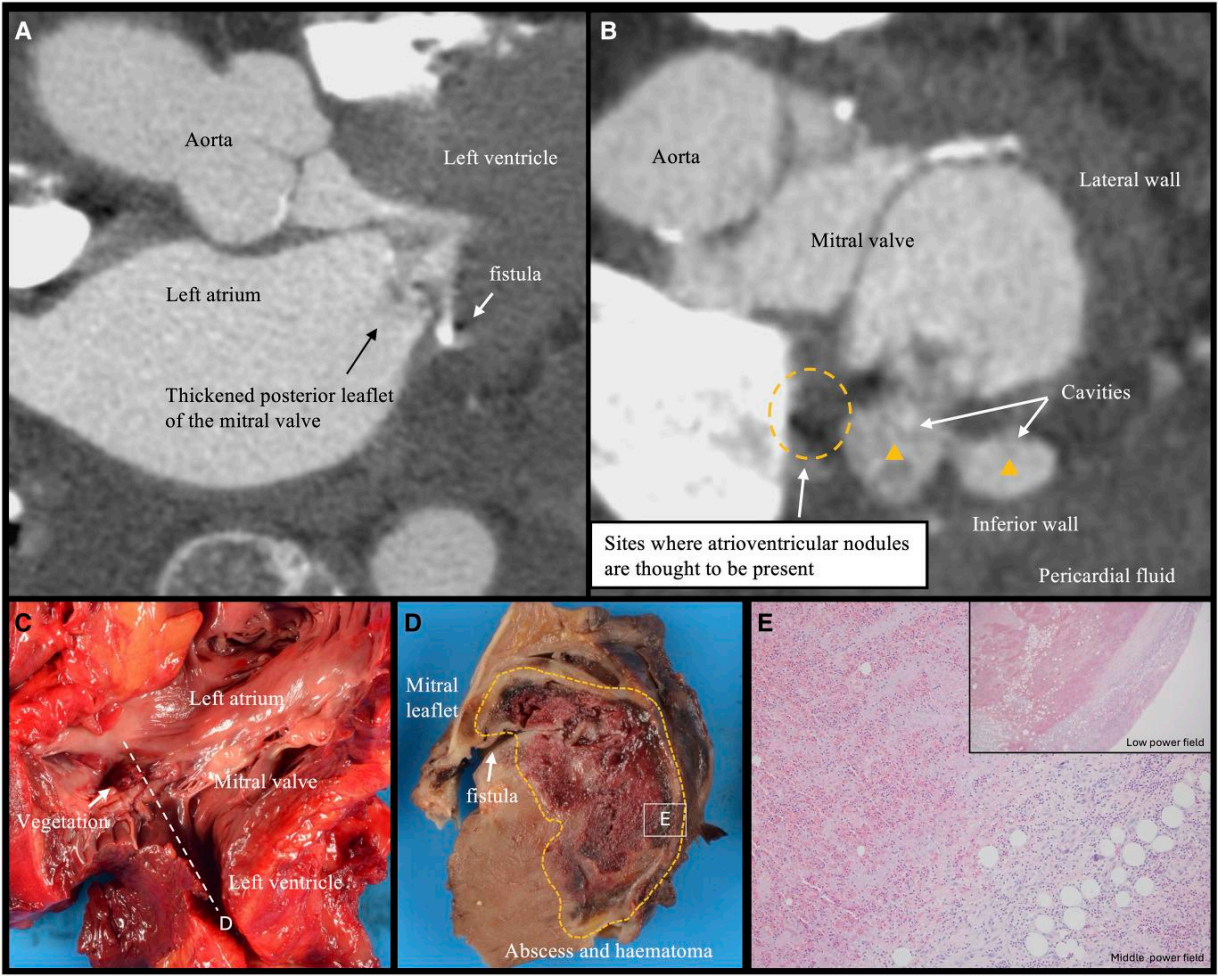
Cardiac computed tomography revealed thickened posterior leaflet of the mitral valve and an abscess within the left ventricular posterior wall in the long-axis view (*A*) and short-axis view (*B*). Gross examination of the left heart (*C* and *D*) demonstrated fistula formation in the lower portion of the posterior apex of the mitral valve and an abscess in the posterior wall. Haematoxylin and eosin staining of the left ventricular free wall (*E*) showed evidence of necrosis and inflammatory infiltration into the myocardial tissue, without clear rupture of the left ventricular wall.

An 87-year-old woman with a history of Type 2 diabetes mellitus was admitted with fever. During her hospitalization, she developed cerebellar infarction and complete atrioventricular block. Transthoracic echocardiography (TTE) revealed a mobile structure on the posterior leaflet of the mitral valve (MV). As transoesophageal echocardiography was contraindicated due to dyspnoea, a cardiac computed tomography (CT) was performed. It demonstrated thickened leaflet of the MV and cavities within the inferior-posterior wall of the left ventricle (*Figure 1*). Blood cultures identified *Staphylococcus aureus*. The heart team recommended cardiac surgery, but the patient declined due to advanced age. Palliative care was initiated, with bedside follow-up conducted through TTE. She succumbed to her illness on the 12th day of hospitalization.

Autopsy revealed a 16 × 9 mm vegetation confined to the posterior leaflet of the MV, accompanied by a left ventricular posterior wall abscess with inflammatory cell infiltration extending to the surrounding tissue, including the atrioventricular node (*Figure 1*). We hypothesized that inflammatory spillover into the conduction system resulted in severe atrioventricular block, while spillover into the pericardial space led to cardiac tamponade.

While transoesophageal echocardiography is strongly recommended for the diagnosis of extravalvular complications of infective endocarditis, it is difficult to perform in high-risk patients in the acute phase.^1,2^ In this case, cardiac CT a high-sensitivity, non-invasive, and rapid modality effectively overcame this limitation and provided an accurate diagnosis.

## Data Availability

The data underlying this article will be shared on reasonable request to the corresponding author.
